# Identification of IFITM3 and MGAT1 as novel interaction partners of BRI3 by yeast two-hybrid screening

**DOI:** 10.3906/biy-1805-47

**Published:** 2018-12-10

**Authors:** İzzet AKİVA, Necla BİRGÜL İYİSON

**Affiliations:** 1 Department of Molecular Biology and Genetics, Faculty of Arts and Sciences Boğaziçi University , Bebek, İstanbul , Turkey

**Keywords:** Wnt/β-catenin signaling, BRI3, SAGE, hepatocellular carcinoma, yeast two-hybrid, IFITM3, MGAT1

## Abstract

BRI3 (brain protein I3) is one of the Wnt/β-catenin pathway target genes as indicated by the results of serial analysis of gene expression (SAGE) and microarray analyses performed in our laboratory. The Wnt/β-catenin signaling pathway is an evolutionarily conserved pathway, which has important functions in early vertebrate development, axis formation, cellular proliferation, and morphogenesis. Previous studies showed that BRI3 expression is upregulated at both mRNA and protein levels upon β-catenin activation by various approaches, such as lithium treatment and overexpression of Wnt ligands in Huh7 (hepatocellular carcinoma) cell lines. Moreover, with regard to the previous literature, BRI3 was found to have a very important role in the TNFα-mediated cell death pathway. In this study, we screened a human liver cDNA library by yeast two-hybrid assay using BRI3 protein as bait, with the aim of finding novel interaction partners of BRI3. Library screening by yeast mating resulted in the identification of three candidate positive clones. Among these, IFITM3 and MGAT1 proteins were confirmed as interaction partners by using cotransformation in yeast cells and coimmunoprecipitation from mammalian cell lines. Considering the poor functional characterization of BRI3 to date, identification of novel BRI3-interacting proteins is an essential first step in determining the action mechanism of BRI3 with respect to the Wnt/β-catenin pathway.

## 1. Introduction

BRI3 (brain protein I3) was originally identified as a 125-amino-acid transmembrane protein that is overexpressed in TNFα-treated L929-murine fibrosarcoma cells (Wu et al., 2003). The blocking of new BRI3 protein synthesis by using BRI3-antisense RNA resulted in increased resistance of these cells to TNF-induced cell death with magnitude greater than 1000-fold. Although the exact action mechanism of BRI3 within the TNFinduced cell death pathway still remains unknown, it is hypothesized that BRI3 synthesis might act as a negative checkpoint of this pathway (Wu et al., 2003).

BRI3 has been selected among the potential Wnt/βcatenin signaling pathway targets based on serial analysis of gene expression (SAGE) screening and an equivalent microarray screening (Kavak et al., 2010) . In order to identify novel transcriptional targets of the Wnt/β-catenin signaling pathway, these transcriptome profile analyses were performed in our laboratory using stable Huh7 (hepatocellular carcinoma) cell lines overexpressing a mutant form of β-catenin, which is degradation-resistant. BRI3 was among the several putative Wnt/β-catenin target genes that were detected with differential expression profiles upon β-catenin induction in the Huh7 cell line. Moreover, lithium treatment of Huh7 cell lines and overexpression of the Wnt ligands in the same cell lines resulted in the upregulation of BRI3 gene expression, as determined by quantitative RT-PCR (Kavak et al., 2010) . The results obtained from luciferase reporter gene assay, in which BRI3 promoter activity was found to be increased due to overexpression of β-catenin, also supported the previous data. Additionally, chromatin immunoprecipitation (ChIP) assays indicated that β-catenin interacts with the BRI3 promoter region in Huh7 cell lines and in mouse liver tissue.

Wnt signaling is an evolutionarily conserved pathway in various organisms from worms to mammals and plays important roles in several biological processes such as development, differentiation, cellular proliferation, morphology, motility, and cell fate. Wnt proteins constitute a family of secreted cysteine-rich glycoproteins that exhibit distinct expression patterns in embryo and adult organisms (Cadigan and Nusse, 1997) . In mammals, 12 distinct Wnt protein families exist, which might induce at least four different pathways: the canonical Wnt/β-catenin/ TCF pathway, and the noncanonical pathways, namely the Wnt/calcium, Wnt/planar cell polarity (PCP), and Wnt/G protein pathways (Wodarz et al., 1998). However, alterations of the canonical Wnt/β-catenin/TCF pathway are implicated in tumorigenesis.

If the Wnt/β-catenin signaling pathway is not activated, cytoplasmic β-catenin levels are kept low through continuous proteasome-mediated degradation, which is controlled by a multiprotein complex containing glycogen synthase kinase 3β (GSK-3β), adenomatous polyposis coli (APC), and axin. In the absence of a Wnt signal, β-catenin is present in the axin complex. In this complex, cytosolic β-catenin, but not the cadherin-bound β-catenin, is continuously phosphorylated, ubiquitinated, and degraded by proteasome (Rubinfeld et al., 1993).

The activation of the Wnt/β-catenin signaling pathway is initiated by binding of a Wnt ligand to the Frizzled receptor (Fz) and low-density lipoprotein receptor-related protein (LRP) 5/6 coreceptor. In this case, Dishevelled (Dsh) inhibits the GSK-3β-dependent phosphorylation of β-catenin in response to the Wnt signal. Consequently, β-catenin is dissociated from the destruction complex and starts to accumulate in the cytosol. The accumulated β-catenin is then translocated into the nucleus, binds to the T-cell factor/lymphoid enhancer factor (TCF/LEF) family of transcription factors, and activates the expressions of several cell cycle- and differentiation-related target genes such as axin, c-myc, and cyclin D1 (Behrens et al., 1996) . In this study, we identify two novel BRI3-interacting proteins, which is an essential first step in determining the action mechanism of BRI3 with respect to the Wnt/βcatenin pathway.

## 2. Materials and methods

### 2.1. Library screening by yeast mating

The Matchmaker GAL4-Yeast Two-Hybrid System (Clontech) was used together with the pretransformed human liver cDNA library (Clontech). A fresh and large colony of the bait strain (AH109 [pGBKT7/Bri3]) was inoculated into 50 mL of SD/-Trp liquid medium and incubated at 30 °C with shaking (230–250 rpm) until the OD600 reached 0.8. The cells were centrifuged at 1000 × g for 5 min and then resuspended in 5 mL of SD/-Trp. In a sterile 2-L flask, a 1-mL aliquot of the library strain was combined with 5 mL of bait strain. Then 45 mL of 2X YPDA liquid medium was added to the flask and incubated at 30 °C for 22 h with slow shaking (50 rpm). After 22 h the cells were centrifuged. The 2-L flask was rinsed twice with 50 mL of 0.5X YPDA. The rinses were then combined and used to resuspend the pelleted cells. The cells were again centrifuged for 10 min and all pelleted cells were resuspended in 10 mL of 0.5X YPDA liquid medium. The mated culture was spread on SD/-Ade/-His/-Leu/Trp plates (200 µL per 150-mm plate). The plates were incubated at 30 °C for 5–6 days.

The yeast host strains used in this study were obtained from Clontech (USA): AH109 (genotype: MATa, trp1-901, leu2-3, 112, ura3-52, his3-200, gal4Δ, gal80Δ, LYS2::GAL1UAS-GAL1TATA-HIS3,GAL2UASGAL2TATA-ADE2,URA3 :: MEL1UAS-MEL1TATA-lacZ) and Y187 (genotype: MATα, ura3-52, his3-200, ade2101, trp1-901, leu2-3, 112, gal4Δ, met–, gal80Δ, URA3 :: GAL1UAS-GAL1TATA-lacZ).

### 2.2. Yeast colony PCR

The identification of the cDNA clone in the prey vector was done by yeast colony PCR and sequencing of the PCR samples. For this purpose, single and fresh colonies were picked with sterile pipette tips and resuspended in 3 µL of NaOH (25 mM) in separate PCR tubes. Liquid nitrogen was used to quick-freeze the samples. Then the samples were placed into the PCR machine and boiled at 95 °C for 10 min. Master mix was prepared using the pACT2FpACT2R primer pair and distributed to the tubes. For the PCR reaction, the following cycling conditions were used: 94 °C for 5 min, 30 cycles [94 °C for 30 s, 55 °C for 30 s, 72 °C for 1 min], 72 °C for 10 min, hot-start at 94 °C.

### 2.3. Cotransformation of competent yeast cells

Clontech’s YeastMaker Yeast Transformation System was used according to the manufacturer’s protocol for all transformations of yeast cells. A PEG/LiAc (polyethylene glycol 3350/lithium acetate)-based method was applied for the preparation and transformation of competent yeast cells. For the cotransformation of the bait and prey vectors, 0.2 µg of each vector was used together with 5 µL of herring testes carrier DNA (10 mg/mL).

### 2.4. Coimmunoprecipitation

HEK293T cells were transfected with plasmids carrying the tagged versions of the genes. First, a 1:1 suspension of protein G agarose bead slurry was washed with icecold lysis buffer three times. Anti-HA antibody diluted in 1X PBS was incubated with 30 µL of protein G agarose beads for 4 h at room temperature by swinging head-overtail. After the incubation, the antibody-bead complexes were washed 3 times with Co-IP lysis buffer at 4 °C by spinning, and then 48 h after transfection adherent cells were washed with ice-cold 1X PBS and then lysed in icecold Co-IP lysis buffer supplemented with 1X protease inhibitor cocktail and 1 mM PMSF. The cells on the plate were harvested using a cell scraper and transferred to an Eppendorf tube. Complete lysis was assured by pipetting the solution several times and incubating on ice for 30 min. The cell lysate was centrifuged at 12,000 rpm for 15 min at 4 °C; 30–40 µL of input was obtained and the remaining supernatant was added to the antibody-bead complex in Eppendorf tubes. The tubes were allowed to swing headover-tail at 4 °C in a cold room overnight. The next day, the antibody-bead-protein complexes were washed three times with Co-IP lysis buffer and once with 1X PBS. The final supernatant was removed and the tubes were stored at –80 °C for later use in SDS-PAGE analysis.

### 2.5. Confocal microscopy

Huh7 cells grown on 18-mm coverslips inside 12-well culture plates were used for imaging with confocal microscopy. The cells were transfected with the plasmids expressing the fluorescent-tagged versions of our proteins of concern using the Turbofect transfection reagent (Thermo Scientific) according to the manufacturer’s instructions. Forty-eight hours after transfection, the growth medium was aspirated and the cells were washed with 1X PBS. Thereafter, 250–300 µL of ice-cold 4% paraformaldehyde was added to the cells in each well. The cells were incubated at room temperature for 20 min without shaking. Then 4% paraformaldehyde was aspirated and the cells were washed with 1X PBS three times. Next, 250 µL of DAPI solution (1 µg/mL) was added to the cells. The cells were incubated with DAPI for 1–2 min and then washed with 1X PBS three times. Using forceps, the coverslips inside the wells were placed onto the slides with 3 µL of mounting medium. After drying for a few minutes, nail polish was applied to intersection areas around the coverslips and the samples were analyzed with the confocal microscope.

## 3. Results

The candidate transcriptional targets of the canonical Wnt/β-catenin pathway were determined previously in our laboratory by using genome-wide microarray analyses and SAGE techniques (Kavak et al., 2010) . BRI3 has been selected as being one of the most prominent targets of Wnt/ β-catenin pathway due to its transcriptional upregulation in hepatocellular carcinoma cells overexpressing the mutant and degradation-resistant form of β-catenin. This upregulation was further supported by experimental evidence coming from q-RT-PCR analyses, ChIP assay, luciferase reporter assay, and treatment of cells with lithium chloride, which leads to the activation of Wnt/βcatenin pathway (Kavak et al., 2010) .

**Figure 1:**
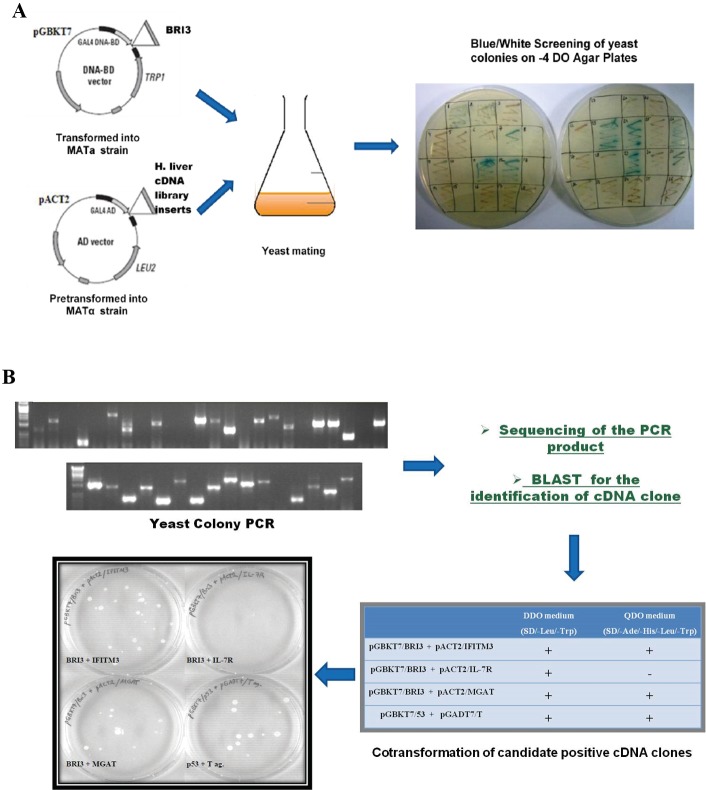
Experimental outline of the yeast two-hybrid assay. Yeast mating and screening of the resulting colonies on nutrient selective media (A). Identification of candidate cDNA clones by colony PCR, sequencing, and confirmation by cotransformation into yeast cells (B).

Yeast two-hybrid assay was performed as a first step in order to determine the interaction partners of the BRI3 protein. For this purpose, a pretransformed human liver cDNA library was used. Yeast mating was performed between the MATa strain (AH109) transformed with the pGBKT7/BRI3 bait vector and the MATα strain (Y187) pretransformed with the human liver cDNA library. The estimated number of independent clones screened by this mating was calculated to be 2.6 × 106. The mated culture was allowed to grow on SD/-Ade/-His/-Leu/-Trp agar plates, so that high stringency was used in order to detect the activation of the reporter genes ADE2 and HIS3. The positive clones obtained from yeast mating were restreaked to single colonies on SD/-Ade/-His/-Leu/-Trp agar plates with X-α-Gal in order to confirm the phenotype. As a result of two-hybrid interactions, in addition to ADE2 and HIS3 reporter genes, MEL1, which encodes the enzyme α-galactosidase, can also be expressed. Yeast colonies that express MEL1 turn blue in the presence of the chromogenic substrate X-α-Gal. Therefore, those single blue colonies were analyzed primarily for being candidate positive colonies (Figure 1A).

The yeast colonies selected in the blue/white screening were subjected to colony PCR analysis in order to identify the corresponding cDNA inserts. PCR products that corresponded to single and clear bands were sequenced and the identities of those cDNA clones were revealed by performing a BLAST search of the raw outputs over various databanks in order to find significant matches. A list of all candidate proteins determined as putative interaction partners is given as supplementary material (Table S1). Several candidates from this list have abundant protein expression levels in the liver cDNA library and are mostly characterized as “sticky” proteins in yeast twohybrid screenings with respect to the previous literature; therefore, these proteins were eliminated as being false positives. Eventually, three candidate cDNA clones (IFITM3, IL-7R, and MGAT1) were determined for further analysis. The vectors containing the cDNA insert of interest were isolated from the corresponding yeast colonies. Then the bait vector together with the vector containing the candidate cDNA clone were cotransformed into the yeast cells for confirmation. The transformed yeast cells were spread on nutrient selective media (SD/-Ade/His/-Leu/-Trp) and tested for their ability to grow into colonies. As a result, cotransformation yielded colonies for two out of three selected cDNA clones (Figure 1B). uhTs, IFITM3 and MGAT1 were determined as candidate interacting partners for BRI3.

**Figure 2:**
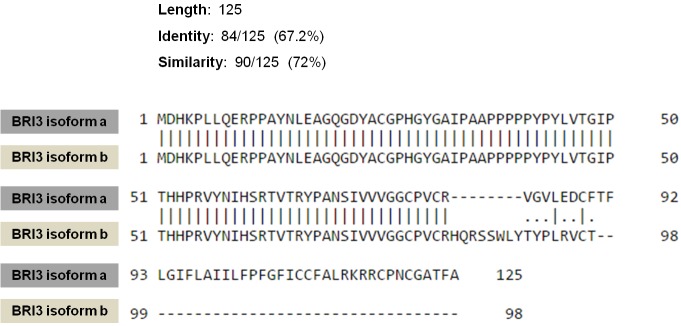
Alignment and comparison of the amino acid sequences for the two isoforms of BRI3. Pairwise sequence alignment was performed using EMBOSS Needle tool.

The BRI3 protein has two isoforms. The a-isoform is 125 amino acids in length. On the other hand, the b-isoform of BRI3 is a 98-amino-acid polypeptide sharing the same N-terminus but has a totally distinct C-terminus compared to the a-isoform (Figure 2). Coimmunoprecipitation has been performed in order to test the interaction of the two BRI3 isoforms with the candidate proteins, which were revealed as the result of yeast two-hybrid assay. For this purpose, the two isoforms of BRI3 were overexpressed in HEK-293T cells together with the candidate proteins IFITM3 and MGAT1, which were cloned into the pcDNA3-HA vector to express the HA-tag fusion constructs. Immunoprecipitation was performed from HEK-293T lysates by using HA-antibody and immunoblotting was done with GFP-antibody in order to detect the GFP-fused versions of BRI3 isoforms. BRI3BP (BRI3 binding protein) was used as a positive control since it is an already known interacting partner of BRI3 protein.

**Figure 3:**
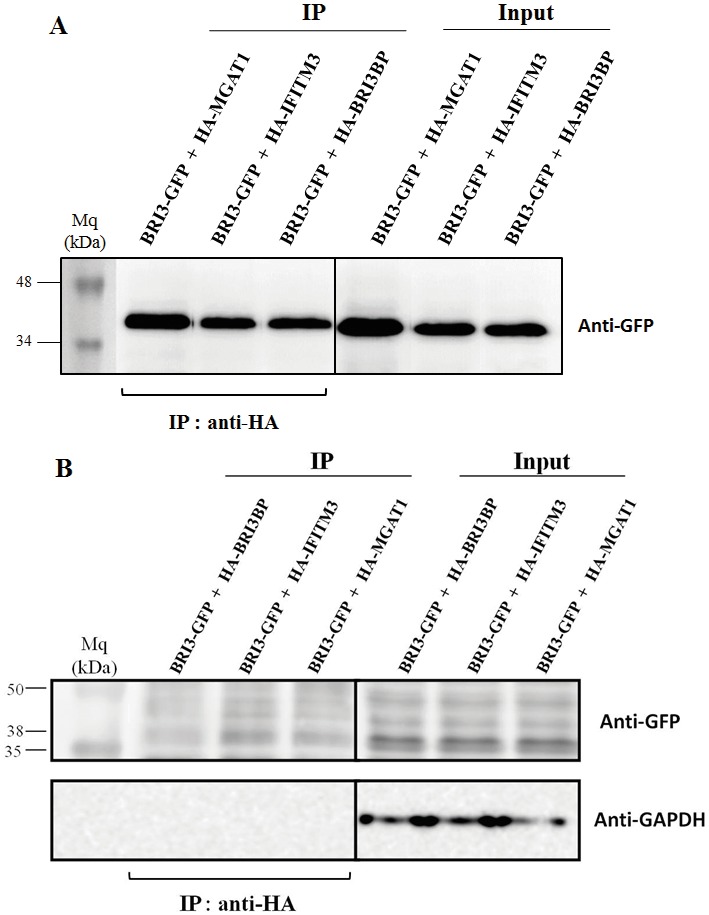
Coimmunoprecipitation of BRI3 a-isoform (A) and b-isoform (B) with the candidate proteins from HEK-293T cells using HA-antibody and immunoblotting with anti-GFP antibody. BRI3BP is used as the positive control for the interaction.

The results of coimmunoprecipitation experiments indicated that the a-isoform of BRI3 is able to interact both with the IFITM3 and MGAT1 proteins (Figure 3A and Figure S1). In contrast, by comparing the protein band intensities, it can be said that the b-isoform of BRI3 demonstrates a much weaker interaction with these two candidate proteins and with the positive control BRI3BP, as well (Figure 3B).

In order to visualize the possible interaction of BRI3 isoforms with the candidate proteins MGAT1 and IFITM3, colocalization assay was performed in the Huh7 hepatocellular carcinoma cell line. For this purpose, GFPtagged BRI3 isoforms were used together with dsREDtagged MGAT1 and IFITM3. The fluorescently tagged proteins were expressed in Huh7 cells and 48 h after transfection the cells were fixed and transferred onto glass slides in order to be imaged with confocal microscopy.

**Figure 4:**
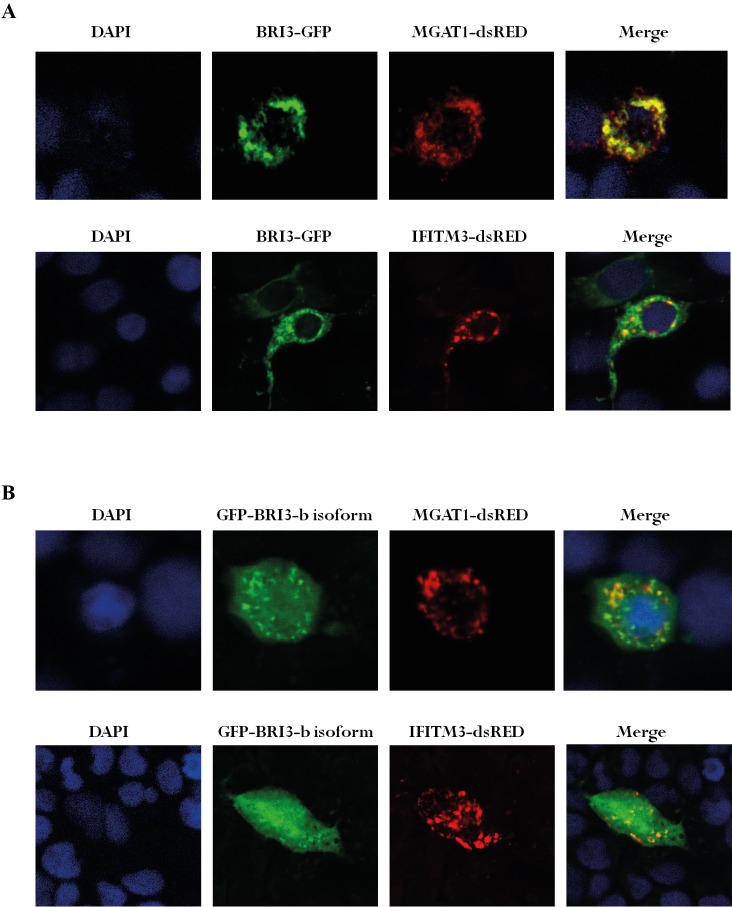
Confocal microscopy images of colocalization assay in Huh7 cells expressing GFP-tagged BRI3 a-isoform (A) and BRI3 bisoform (B) together with either MGAT1-dsRED or IFITM3-dsRED constructs.

As a result of the colocalization assay, the a-isoform of BRI3 can be seen to colocalize with both MGAT1 and IFITM3, especially in the perinuclear area and possibly inside the organelles such as the Golgi apparatus or ER (Figure 4A). On the other hand, the b-isoform of BRI3 is mostly seen to be uniformly distributed throughout the cell as being both in the cytoplasm and nucleus, rather than having a specific subcellular localization. In that case, a lower extent of colocalization is observed for the b-isoform of BRI3 with either MGAT1 or IFITM3 (Figure 4B).

## 4. Discussion

The canonical Wnt/β-catenin pathway is a highly conserved signaling pathway; it is involved in various differentiation events during embryonic development, such as axis formation, cellular proliferation, differentiation, and morphogenesis. β-Catenin is considered to be the key molecule in this pathway. In addition to its functions in early vertebrate development, the Wnt/β-catenin pathway has the potential to initiate tumor formation when aberrantly activated. Those characteristics make the Wnt/ β-catenin signaling pathway itself and its targets important subjects in cancer research fields, since genes regulated by this pathway are potential drug and gene therapy targets. Therefore, with the purpose of identifying novel transcriptional targets of the Wnt/β-catenin pathway, SAGE and microarray screenings were carried out in our laboratory. Using these techniques, several genes were determined to be either upregulated or downregulated significantly in response to mimicking the active status of the Wnt/β-catenin pathway.

BRI3 was found to be one of the novel transcriptional targets of the Wnt/β-catenin signaling pathway. It was selected from the SAGE screening results due to the fact that overexpression of the degradation-resistant β-catenin mutant (S33Y-β-catenin) resulted in significant upregulation of BRI3. Supporting data were obtained from lithium treatment of Huh7 cell lines, luciferase reporter assay, overexpression of Wnt ligands, and ChIP assay by using the anti-β-catenin antibody (Kavak et al., 2010) .

BRI3 has been poorly characterized so far, as can be judged from the current literature. Its function and action mechanism are largely unknown. Therefore, we aimed to provide clues about the functional relevance of BRI3 for the Wnt/β-catenin pathway. As a first step in our study, we intended to discover novel interaction partners of the BRI3 protein in order to shed light on the action mechanism of BRI3. Yeast two-hybrid assay was employed for this purpose. A human liver cDNA library was screened using BRI3 protein as bait. Following the two-hybrid library screening by yeast mating, colonies were selected on highstringency growth media (-4 dropout media lacking Ade/ His/Leu/Trp amino acids) (Figure 1A). Identification of the candidate interaction partners corresponding to the positive colonies was performed by means of yeast colony PCR and sequencing (Figure 1B). We were able to identify MGAT1 (mannosyl α-1,3-glycoprotein β-1,4-Nacetylglucosaminyl transferase) and IFITM3 (interferon induced transmembrane protein 3) as candidate positive interactors, which were confirmed by cotransformation into yeast cells. On the other hand, the third possible candidate protein, IL-7R, was eliminated after yeast cotransformation due to lack of colony growth on highstringency selective media.

With the purpose of verifying the interactions, further experiments were performed using the two isoforms of BRI3, which result from an alternative splicing event. Alignment of the amino acid sequences of the two BRI3 isoforms suggest that the N-terminals are the same; however, the BRI3 a-isoform has a distinct C-terminus compared to the shorter BRI3 b-isoform (Figure 2).

Coimmunoprecipitation was performed in order to test for the interaction of these BRI3 isoforms with the candidate binding partners obtained from the yeast twohybrid assay. A strong positive interaction was determined for the a-isoform of BRI3 with candidate proteins MGAT1 and IFITM3 (Figure 3A). However, in the case of the shorter b-isoform of BRI3, very faint protein bands can be observed in coimmunoprecipitation, suggesting a much weaker interaction of the b-isoform with these two candidate proteins (Figure 3B). BRI3BP was used as the positive control for interaction since it was the only known protein interactor of BRI3 (Yamazaki et al., 2007). A further point is that, in the case of the BRI3 isoform-b, we could not detect any band corresponding to isoform-b even for the input samples in western blotting. The possible reason for this is the failure of the BRI3 antibody to recognize this shorter BRI3 isoform due to the differences in amino acid sequences of these two isoforms, especially in their C-terminals.

For further confirmation, a colocalization assay was performed in Huh7 cell lines by expressing the fluorescent- tagged proteins in order to visualize their subcellular localizations. The results indicate an intense colocalization of BRI3 with MGAT1, especially in the perinuclear area of Huh7 cells, which might be inside an organelle such as the Golgi apparatus or ER (Figure 4A). In fact, a previous study was carried out to determine the subcellular localization of BRI3 (Wu et al., 2003). The results obtained from this study suggest that the BRI3-GFP fusion protein localizes in the lysosomes within the cell and the function of BRI3 may be related to lysosomes. Colocalization was also observed between the fluorescent-tagged proteins of BRI3 and IFITM3, albeit to a lower extent (Figure 4A). On the other hand, the BRI3 b-isoform appears to be distributed almost uniformly throughout the cells, including both the cytosol and nucleus, and additionally a very low level of colocalization can be observed between the BRI3 b-isoform and candidate binding partners MGAT1 and IFITM3 (Figure 4B). This observation prompts us to hypothesize that the BRI3 protein might have a specific localization signal sequence in its C-terminus, which is not present in the shorter b-isoform.

MGAT1 is known to code for an enzyme essential for the synthesis of hybrid and complex N-glycans. The finding of MGAT1 as an interacting partner of BRI3 can be regarded as promising in the sense that MGAT1 is also one of the genes that exhibit differential expression levels in response to β-catenin activation, as was demonstrated in the initial SAGE analysis. Furthermore, our recent experiments indicated that MGAT1 is upregulated at both mRNA and protein levels in response to β-catenin activation by various approaches. Thus, MGAT1 can be defined as a novel transcriptional target of the Wnt/βcatenin signaling pathway (Akiva et al., 2018) .

According to the previous literature, IFITM3 has been identified as a new molecular marker in human colorectal tumors and it has been stated that IFITM3 gene expression is controlled by Wnt/β-catenin signaling in mouse and human intestinal epithelium (Andreu et al., 2006) . Furthermore, the results of a more recent study indicated elevated IFITM3 expression in colon cancer cells compared to normal colon cells. The data obtained from this study suggest that IFITM3 plays an important role in early colon cancer development (Fan et al., 2008) .

In this study, we aimed to determine the novel interaction partners of BRI3 by yeast two-hybrid assay. In the course of this work, we have identified and confirmed MGAT1 and IFITM3 as binding partners of BRI3. Furthermore, all these three proteins are regulated by Wnt/β-catenin signaling. The functional significance of these novel interactions will be among the most important subjects of future experiments.

## Supplementary Material

List of proteins determined as putative interaction partners by yeast two-hybrid screening. “Sticky” proteins with abundant expression levels in cDNA library are determined as false positive.
