# Supplementary material for: Identification of IFITM3 and MGAT1 as novel interaction partners of BRI3 by yeast two-hybrid screening
Source: Turk J Biol. 2018 Dec 10;42(6):463–70. doi: 10.3906/biy-1805-47 (PMC6451842; doi:10.3906/biy-1805-47)
Supplement: List of proteins determined as putative interaction partners by yeast two-hybrid screening. “Sticky” proteins with abundant expression levels in cDNA library are determined as false positive. [file turkjbio-42-463-s001.pdf]

Table S1. List of proteins determined as putative interaction partners by yeast two-hybrid screening. “Sticky” proteins with abundant expression levels in cDNA library are determined as false positive.

Gene symbol	Protein name	Description
IL-7R	Interleukin 7 receptor	Candidate interaction partner
MT2A	Metallothionein 2A	False positive
IFITM3	Interferon induced transmembrane protein 3	Candidate interaction partner
AOX1	Aldehyde oxidase 1	False positive
HEBP2	Heme-binding protein 2	False positive
VTN	Vitronectin	False positive
ALDOB	Aldolase B, fructose bisphosphate	False positive
HPX	Hemopexin	False positive
MGAT1	Mannosyl (α-1,3-)glycoprotein β-1,4-N-acetylglucosaminyltransferase	Candidate interaction partner
MT1X	Metallothionein 1X	False positive
TFR2	Transferrin receptor 2	False positive
TIMP1	TIMP metallopeptidase inhibitor 1	False positive
APOH	Apolipoprotein H (beta-2-glycoprotein I)	False positive
C8A	Complement component 8, alpha polypeptide	False positive
LRPAP1	Low density lipoprotein receptor-related protein associated protein 1	False positive
SLC2A4	Solute carrier family 2 (facilitated glucose transporter)	False positive
TM7SF3	Transmembrane 7 superfamily member 3	False positive

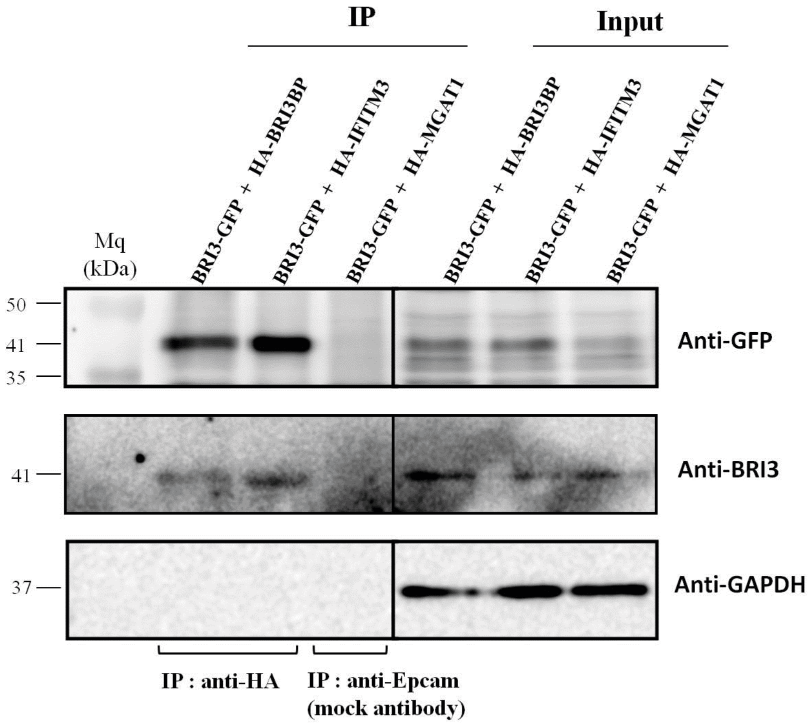

Figure S1. Coimmunoprecipitation of BRI3 a-isoform with the candidate proteins MGAT1 and IFITM3 from HEK-293T cells using the HA-antibody and immunoblotting with anti-GFP, anti-BRI3, and anti-GAPDH antibodies. BRI3BP is used as positive control for the interaction. Anti-Epcam antibody is used as a mock antibody (negative control for immunoprecipitation).
